# Career women's mental wellbeing in the era of population decline: the effects of working environment and family environment on the mental wellbeing

**DOI:** 10.3389/fpubh.2024.1462179

**Published:** 2024-09-25

**Authors:** Bowen Zhou, Xuchen Wu, Ruixue Ge, Dongni Zhuo

**Affiliations:** ^1^School of International Economics and Trade, Jilin University of Finance and Economics, Changchun, China; ^2^College of Education, University of Washington, Seattle, WA, United States

**Keywords:** era of population decline, career women, mental wellbeing, working environment, family environment

## Abstract

**Introduction:**

In recent years, it has become increasingly evident that the population in many countries has been declining. China, which was previously the world's most populous nation and is often categorized as an emerging economy, officially entered an era of population decline in 2022. The advent of this era has make China's economic development more uncertain and aging of population more pronounced. To address the population decline, the Chinese government implemented the “Three-Child Policy” to encourage childbirth, aiming to reverse the negative population growth. However, this policy has not achieved the expected goals. Instead, it has increased the pressure on women to bear children, particularly for career women, where such pressure may conflict with their existing work and family environments, subsequently affecting their mental wellbeing.

**Methods:**

A survey was conducted to investigate the mental wellbeing status of career women in Changchun City, Jilin Province, Northeast China. It analyzes the impact of working and family environments on the mental wellbeing of these women.

**Results:**

Based on the survey, this study draws five conclusions: **A**. The mental wellbeing status of career women varies across different ages, industries, and childbirth statuses. **B**. The perceived adverse impact of childbirth on the working environment may negatively affect the mental wellbeing of career women. **C**. The perceived adverse impact of childbirth on the family environment may negatively affect the mental wellbeing of career women. **D**. Career women are not satisfied with the effectiveness of current policies in protecting women's rights. **E**. Compared to working environments, there is a greater demand for career women in the family environments, particularly in reducing various family burdens.

**Discussion:**

The pro-natalist policies introduced in response to negative population growth can worsen the mental wellbeing of career women, while the deterioration of their mental wellbeing could further accelerate population decline. Given the current challenges, this study suggests that effectively improving the mental wellbeing of career women requires building psychological resilience among childless career women, reducing the burden of family on career women, and continuously improving policies and regulations that protect the rights of career women.

## 1 Introduction

According to the statistics from the Population Division of the United Nations, the global rate of natural change has shown a declining trend from 2000 to 2021.^1^ This decline is particularly pronounced in high-income countries, where negative population growth is more evident. As of 2021, more than half of these countries, specifically 19, have experienced population decline. Meanwhile, several emerging economies have also begun to enter an era of population decline. From 2000 to 2021, India, as the world's second-most populous country during that period, saw its natural population growth rate drop from 18.3% to 7.0%, indicating a trend toward population decline. China, previously the world's most populous nation and a major emerging economy, has also been significantly affected by negative population growth and officially entered an era of population decline in 2022. China's demographic challenges are closely linked to its historical population policies. The One-Child Policy, implemented from the late 1970s to 2015, was designed to curb the rapidly growing population, given the economic conditions and social issues of that time (1, 2). This policy, while effective in slowing population growth, has led to a significant imbalance in the population structure, with an aging population and a shrinking workforce becoming increasingly evident (3). The policy resulted in what is commonly referred to as the “4-2-1” family structure, where one child is responsible for supporting two parents and four grandparents. In response to these demographic shifts, the Chinese government introduced the Two-Child Policy on January 1, 2016, allowing families to have two children in an effort to mitigate the aging population problem. However, this policy had limited success; according to data from China's National Bureau of Statistics, the natural population growth rate briefly increased by 1.6% in 2016 compared to 2015 but continued to decline thereafter.^2^ As a result, the government further relaxed birth restrictions by implementing the Three-Child Policy in May 2021. The implications of these policies have been profound, particularly for career women. With the gradual relaxation of birth control policies, career women face increasing pressure to balance professional responsibilities with family duties. Not only are they expected to contribute to the household income, but they also bear a significant portion of family caregiving responsibilities, which may impact their mental wellbeing and career progression.

According to the “Green Paper on the Mental Wellbeing of Chinese Career Women, 2019,” approximately 30% of women reported feeling “anxious and depressed from time to time,” while 7% indicated that they “are always in a state of anxiety or depression,” highlighting the increasing mental wellbeing challenges among career women.^3^ Compared to non-career women, career women face unique and multifaceted pressures that arise from their dual roles in both professional and domestic spheres. As a significant part of the workforce, career women not only contend with workplace demands, such as job performance pressure, promotion competition, and economic uncertainties, but they also shoulder substantial family responsibilities, including childcare and eldercare. This study focuses exclusively on career women due to the distinct and heightened challenges they face in balancing work and family life. The pressures stemming from these dual roles may contribute to a higher risk of mental wellbeing issues, making this demographic particularly vulnerable (4). While a comparative analysis with non-career women could offer additional insights, the unique stressors and societal expectations placed upon career women warrant an in-depth and focused examination in this research. In the working environments, because of economic uncertainties, companies may reduce expenses by laying off employees and intensified the workload of remaining staffs. It has further deteriorated the work environments (5). In the family environments, because of the population aging and increasing life expectancy, a family may have to fully support up to four older adults simultaneously (6). It could significantly increase the families' financial burden. Moreover, influenced by the Three-Child Policy and traditional Chinese beliefs, some women are expected by their families to take on more childbirth responsibilities. Thus, maintaining mental wellbeing amidst the pressures of a challenging work environment and demanding family responsibilities is a common dilemma faced by career women in China.

This study, in the context of China's negative population growth, examines the impact of the working environments and family environments on the mental wellbeing of career women. This research not only enriches the understanding of women's mental wellbeing but also provides valuable insights for other emerging economies facing similar population decline issues. Additionally, this study holds significant practical implications for social stability, corporate development, and family happiness. From a perspective of society, investigating the mental wellbeing of career women in the context of population decline helps improve the effectiveness, rationality, and practicality of population policies and economic policies. For a perspective of employer, there is a direct relation between employees' mental wellbeing and their performance. Maintaining the mental wellbeing of career women can enhance operational efficiency and effectiveness (7). From a perspective of family, women's wellbeing is closely tied to family's stability; their mental wellbeing significantly impacts their children's and partners' physical and psychological wellbeing (8).

## 2 Literature review

Initially, population decline was measured by the positive or negative natural growth rate (9, 10). As research progressed, Wang Feng and his colleagues (11) argued that using only the natural growth rate to measure population changes is not comprehensive. They suggested excluding the influence of the population age structure and using the intrinsic natural growth rate to measure population changes. The causes of population decline can be categorized into exogenous and endogenous factors. Exogenous causes include events that distort social development, such as wars, plagues, and famines (12). Endogenous causes stem from factors such as increased life expectancy, an aging age structure, a long-term decline in birth rates, and shifts in social ideologies (13). China's population decline is mainly attributed to endogenous factors (14). Population decline inevitably exacerbates population aging and the imbalance in the age structure. To encounter these issues, the government would implement policies to encourage childbirth. However, such pro-natalist policies increase the costs for companies to hire female employees, potentially reducing their willingness to hire women and leading to greater employment discrimination against career women (15). Moreover, in Chinese society, women's mental wellbeing could be impacted by the deeply entrenched belief that women should shoulder more family responsibilities. On one hand, this belief leads many women to accept these duties as their obligation. On the other hand, they find it increasingly difficult to cope with the ever-growing family obligations. This conflicting mindset often triggers mental wellbeing issues (16, 17). The employment pressure faced by career women in the workplace, combined with the expectations for childbirth and family obligations, can lead to varying degrees of mental health issues, thereby affecting their willingness to have children (18). Additionally, factors such as pessimism about economic prospects, pressure related to children's education, and the burden of high mortgage payments further contribute to the decline in women's willingness to have children (19). As a result, the government may introduce more policies to stimulate childbirth. However, these policies often overlook the employment and family pressures that career women endure, and additional pro-natalist policies may exacerbate the current predicament faced by career women (20).

With the progress of society, women have increasingly become an indispensable labor force in the work. Although career women today bear the majority of family and work responsibilities, they often do not receive adequate recognition from either their families or work. This leads to poorer mental wellbeing outcomes for career women compared to men (21). Wang et al. (22) conducted a mental wellbeing survey using the Symptom Checklist-90 (SCL-90) on women from 10 industries across 24 provinces and regions in China. The results showed that over one-third of the participants exhibited tendencies toward mental wellbeing issues. According to the Global Burden of Disease study, mental wellbeing issues have become significant factors affecting life expectancy and contributing to various diseases. Anxiety and depression are the primary causes of deteriorating mental wellbeing (23). Clinical research also indicates that women are more likely to be diagnosed with depression and anxiety compared to men (24). Yan et al. (25) investigated the mental wellbeing status of career women in Shanghai and found that the rates of people with anxiety and depression were 48.3% and 70.3%. Lv et al. (26) conducted a mental wellbeing survey on women in various professions in northwest China and discovered that female workers scored the highest for anxiety and depression. Gao et al. (27) found that nearly half of the participants in their study of career women in different communities showed mild to severe depression. To improve the mental wellbeing of career women, scholars have conducted various studies. Lv et al. (28) found that career women experience significant psychological stress during pregnancy, and psychological counseling and interventions through the mobile application WeChat can alleviate this stress. Additionally, for specific occupational groups such as female veterans, it is recommended to hire more female healthcare providers to meet their mental wellbeing needs (29).

Different working environments can have different impacts on employees' mental wellbeing. Factors such as organizational structure, work processes, and compensation systems influence employees' mental wellbeing (30). The “Three-Child Policy” may result in lower salaries and fewer promotion opportunities for career women compared to men, and the income gap between the two may gradually widen. Low-income career women are more prone to mental wellbeing issues, often stemming from domestic violence, work discrimination, and parenting pressures (31, 32). At home, women need to invest more time and energy in childbirth and nurture. It could be difficult for them to balance these responsibilities with their work. Consequently, career women are more susceptible to mental wellbeing issues during pregnancy (33, 34). Empirical results indicate that work-family conflict increases the likelihood of depression among career women (35).

Currently, there is plenty of studies on the mental wellbeing of career women. Many scholars have explored the impact of working or family environments on their mental wellbeing and offered various perspectives. However, there remains a gap examining the impacts within the context of population decline in emerging economies. Based on this, this study aims to answer the following questions: **A**. What factors influence the mental wellbeing of career women? **B**. Are the adverse effects of population decline on the working or family environments of career women contributing to the deterioration of their mental wellbeing? **C**. Do career women understand or are they satisfied with the current policies protecting women's rights?

## 3 Materials and methods

### 3.1 Participants

The survey was distributed in several districts of Changchun, Jilin Province, located in Northeast China, including Nanguan, Kuancheng, Chaoyang, Erdao, Lvyuan, Shuangyang, and Jiutai. The choice of this region for the survey is based on two reasons:

A. **Representative Population Decline:** According to data from the National Bureau of Statistics of China, Jilin Province was the third province in China to experience population decline, making it a representative area for studying this phenomenon.
B. **Comprehensive Industrial Structure:** Changchun, the capital city of Jilin Province, is also one of the central cities in Northeast China and a significant industrial base city. With a comprehensive industrial structure and a relatively advanced economy, this city provides a setting to study the mental wellbeing of career women across various industries.

In this study, we selected participants aged 20 to 45. The lower age limit of 20 was chosen because women cannot marry before this age by Chinese law. The upper age limit of 45 was selected because women beyond this age may be no longer in their prime childbearing years and are less affected by issues related to childbirth. The 20 to 45 age range is significant as it encompasses the period when most women choose to have children and are also progressing in their careers. Therefore, this age range is particularly relevant for investigating the mental wellbeing of career women in the context of population decline. Understanding their mental wellbeing and needs is essential for this study.

The survey was distributed both online and offline in Changchun. A total of 793 surveys were collected, and 688 surveys were deemed valid, accounting for 86.76% of the total.

### 3.2 Survey design

The survey consists of three sections. The first section gathers basic information, including the participant's age, industry, education level, salaries, and marital status. This section aims to collect essential demographic data about the participants.

The second section of the survey focuses on the participants' mental wellbeing status. This study employs the Simplified Scale for Depression and Anxiety Screening (SSADS) to measure the mental wellbeing status of career women (36, 37). This scale is formulated by the Self-Rating Anxiety Scale (SAS-20 items), the Self-Rating Depression Scale (SDS-20 items), and the Symptom Checklist-90 (SCL-90). Specifically, SCL-90 incorporates 13 items related to depression and 10 items related to anxiety. As a result, the scale has good reliability and validity, and it is suitable for screening depression and anxiety among career women in various industries. SSADS comprises 14 items related to two factors: depression (9 items) and anxiety (5 items). Each items is scored on a scale of 1 to 5, with higher scores indicating more severe symptoms. The participants' mental wellbeing status is assessed using both the mean factor scores and the total scale score. The mean factor score is calculated by dividing the total score for the relevant items by the number of items. If the mean factor score exceeds 2, it is considered positive for that factor, indicating that the individual may have anxiety or depression. The total scale score is the sum of all item scores, with a maximum possible score of 70. A total score exceeding 25 is considered positive, indicating the presence of mental wellbeing issues.

For the reliability and validity tests of the 688 valid questionnaires collected in this study, the results are as follows:

**Reliability test:** The overall Cronbach's α coefficient of the questionnaire was 0.939, with the Cronbach's α coefficient for the depression factor being 0.939 and for the anxiety factor being 0.955, all of which passed the reliability test.
**Validity test:** The overall KMO (Kaiser-Meyer-Olkin) coefficient of the questionnaire was 0.956, with a Bartlett's sphericity test value of 8,889.895 (*P* < 0.001). The KMO coefficient for the depression factor was 0.946, with a Bartlett's sphericity test value of 4,944.502 (*P* < 0.001), and the KMO coefficient for the anxiety factor was 0.884, with a Bartlett's sphericity test value of 3,123.786 (*P* < 0.001).

The third section of the survey investigates the impact of working and family environments on the mental wellbeing status of career women. This section is divided into three groups:

A. **Impact of working environment:** The first group focuses on the impact of the working environment on the mental wellbeing status of career women. Specifically, it aims to investigate whether childbirth negatively affects the existing working environments. And in turn, adverse effects worsen the mental wellbeing status of career women.
B. **Impact of family environment:** This group explores the impact of the family environment on the mental wellbeing status of career women. Specifically, it aims to investigate whether childbirth negatively affects the existing family environments. And in turn, adverse effects worsen the mental wellbeing status of career women.
C. **Assessment of satisfaction and needs:** This group assesses the satisfaction and needs of career women regarding their current situation. It examines their awareness and satisfaction with current policies, as well as their needs in the family, workplace, and society.

Additionally, the survey includes two sets of surveys designed for women who have not had children and those who have. This allows for a comparison of how different childbearing statuses affect the working and family environments and, consequently, the mental wellbeing status of career women.

### 3.3 Descriptive statistics

As shown in Table 1, there is a total of 688 participants in this survey, with 35.02% experiencing mental wellbeing challenges. This figure aligns with the data mentioned earlier from the “*Green Paper on the Mental Wellbeing of Chinese Career Woman, 2019”*, which reported that around 30% of Chinese career women have mental wellbeing issues. However, it is notable that the data from the Green Paper was collected in 2019, before the COVID-19 pandemic. Currently, with increasing economic uncertainties in China and the additional pressures from the “Three-Child Policy”, the mental wellbeing of career women continues to deteriorate. The survey was conducted in Northeast China, a region known for having the highest happiness index for women in the country (38). Despite this, one-third of the career women surveyed face mental wellbeing challenges, which means that the mental wellbeing status of Chinese career women could be a concern.

**Table 1:** Descriptive analysis of the basic information of the participants.

**Statistical content**	**Variables**	**Number**	**Proportion**
Portion of positive test results	-	241	35.02%
Age	20–25	56	8.14%
	26–30	99	14.39%
	31–35	180	26.16%
	36–40	216	31.4%
	41–45	137	19.91%
Industry	Financial	74	10.76%
	Education	95	13.81%
	Healthcare	82	11.92%
	Government agencies	63	9.15%
	Cultural and arts	29	4.22%
	Mechanical manufacturing	246	35.76%
	Other	99	14.38%
Education level	Primary school education	4	0.58%
	Middle school education	39	5.67%
	High school/Vocational school degree	59	8.58%
	Associate degree	128	18.6%
	Bachelor's degree	330	47.97%
	Graduate degree or higher	128	18.6%
Monthly income	< 1,300	12	1.74%
	1,300–2,200	19	2.76%
	2,200–3,400	132	19.19%
	3,400–5,000	158	22.97%
	5,000–9,000	252	36.63%
	>9,000	115	16.72%
Marital status	Unmarried	169	24.56%
	Married	494	71.8%
	Divorced	24	3.49%
	Widowed	1	0.15%
Actual number of children raised	0	225	32.7%
	1	383	55.67%
	2	78	11.34%
	3	1	0.15%
	>3	1	0.14%
Expected number of children to be raised	0	141	20.49%
	1	344	50%
	2	180	26.16%
	3	9	1.31%
	>3	14	2.04%

According to the basic information reported in Table 1, the age distribution of the participants is reasonable: 8.14% are aged 20–25, 14.39% are aged 26–30, 26.16% are aged 31–35, 31.4% are aged 36–40, and 19.91% are aged 41–45. The industry distribution of the surveyed participants closely aligns with that of women in Jilin Province, as reported in the Jilin Statistical Yearbook 2023. Specifically, 10.76% work in the financial industry, 13.81% in education, 11.92% in healthcare, 9.15% in government agencies, 4.22% in cultural and arts sectors, 35.76% in mechanical manufacturing, and 14.38% in other industries. This distribution helps us gain deeper understanding into the impact of age and industry on the mental wellbeing of career women.

The majority of the participants have an education level of associate degree or higher, accounting for 85.17%. Specifically, 18.6% have a graduate degree or higher, 47.97% have a bachelor's degree, 18.6% have an associate degree, 8.58% have a high school/vocational school degree, 5.67% have a middle school education, and 0.58% have a primary school education. Based on previous studies on women's income in Northeast China, this survey categorizes women's monthly income into six levels: very low, low, lower-middle, middle, upper-middle, and high (39). Among the participants, 76.31% have an income above the middle level. Specifically, 16.72% earn over 9,000 RMB, 36.63% earn between 5,000 and 9,000 RMB, 22.97% earn between 3,400 and 5,000 RMB, 19.19% earn between 2,200 and 3,400 RMB, 2.76% earn between 1,300 and 2,200 RMB, and 1.74% earn between 0 and 1,300 RMB. Regarding marital status, 71.8% of the participants are married, 24.56% are unmarried, 3.49% are divorced, and 0.15% are widowed. The high proportion of married women in the survey sample helps provide a more accurate study of the impact of working and family environments on the mental wellbeing of career women.

## 4 Results

### 4.1 There are differences in the mental wellbeing of career women across various ages, industries, and childbirth statuses

#### 4.1.1 Mental wellbeing challenges are most pronounced among career women aged 31–35

As shown in Table 2, the mental wellbeing status of career women varies significantly across different age groups. According to the chi-square test results shown on the right side of Table 2, there is a significant difference in the mental wellbeing levels among career women of different age groups. Among these, the highest portion of positive test results was observed among women aged 31–35 at 41.11%, and lowest was observed among women aged 41–45 at 21.90%. The highest portion of positive test results among women aged 31–35 can be attributed to two main reasons. Firstly, women in this age group are often at a critical stage of their professional development. They have to face significant work competition and pressure. Secondly, nearly 70% of women in this age group are mothers. They need to invest substantial time and energy in caring for their children and managing household chores. Additionally, they face multiple financial pressures, including mortgage, car loans, and children's education expenses. These combined stresses result in highest levels of anxiety and depression among career women aged 31–35. Conversely, women aged 41–45 exhibit the best mental wellbeing may be primarily because women in this age group have typically reached a stable phase in their careers. Their positions and incomes are relatively stable, and they face less working stress. Additionally, they have accumulated extensive professional experiences and skills, enabling them to better handle challenges from working environments. Secondly, their children are usually in their teenage or adult years and are becoming increasingly independent. They no longer need to invest significant time and energy in taking care of them. Finally, women aged 41–45 generally have more mature psychological coping mechanisms and more effective stress management skills. They are better able to manage their emotions and mental wellbeing.

**Table 2:** Mental wellbeing status of career women across different age groups.

**Statistical content**	**Variables**	**Number**	**Portion of positive test results**	**Positive rate of depression**	**Positive rate of anxiety**	**Chi-square test**		
						**DF**	**Chi-value**	***P*** **-value**
Age	20–25	56	28.57%	30.36%	17.86%	4	15.985	0.003
	26–30	99	37.37%	36.36%	16.16%			
	31–35	180	41.11%	42.22%	20.56%			
	36–40	216	38.89%	40.28%	22.22%			
	41–45	137	21.90%	25.55%	13.87%			

#### 4.1.2 Mental wellbeing challenges are more severe among career women in education, healthcare, and machinery manufacturing

As shown in Table 3, the mental wellbeing status of career women in the education, healthcare, and machinery manufacturing sectors exhibits more positive test results, at 44.21%, 40.24%, and 38.62%, respectively. According to the chi-square test results shown on the right side of Table 3, there is a significant difference in the mental wellbeing levels among career women across different industries. The highest positive rate in the education sector may be attributed to the dual responsibilities from family and work, which can potentially lead to increased anxiety and depression. This interpretation aligns with findings from previous studies, which suggest that the combined pressures of professional and domestic duties can have a significant impact on mental wellbeing (40). On one hand, educators frequently take heavy workloads and must take their personal time for planning lessons, grading assignments, and addressing various student issues. Their inability to effectively separate work and personal time often results in anxiety and depression (41). On the other hand, compared to other industries, men in China are more likely to choose teachers as their spouses, which is mainly because teaching work offer flexible schedules and long summer and winter vacations. Consequently, women in the education industry are expected to invest more time and energy in caring for children and managing family responsibilities. For women in the healthcare industry, the high positive rate may be associated with the intense nature of their work. Professionals in this industry need to treat a large number of patients daily and handle emergencies frequently. They have limited discretionary time and face immense work pressure (42). In the machinery manufacturing industry, many positions require highly repetitive tasks, which can easily lead to psychological fatigue. Additionally, as the industry is traditionally male-dominated, women may encounter gender discrimination and bias to career advancement. These factors contribute to increased levels of anxiety and depression among women in this industry.

**Table 3:** Mental wellbeing status of career women in different industries.

**Statistical content**	**Variables**	**Number**	**Portion of positive test results**	**Positive rate of depression**	**Positive rate of anxiety**	**Chi-square test**		
						**DF**	**Chi-value**	***P*** **-value**
Industry	Financial	74	28.38%	32.43%	16.22%	6	22.999	0.000
	Education	95	44.21%	44.21%	20.00%			
	Healthcare	82	40.24%	43.90%	19.51%			
	Government agencies	63	17.24%	17.24%	10.35%			
	Cultural and arts	29	20.69%	27.59%	6.90%			
	Machinery manufacturing	246	38.62%	39.43%	26.02%			
	Other	99	29.32%	29.32%	10.53%			

#### 4.1.3 Mental wellbeing challenges are most significant among married career women with two children

As shown in Table 4, compared to the mental wellbeing status of career women with different childbirth statuses, married women with two children exhibit the highest portion of positive test results at 42.67%. According to the chi-square test results shown on the right side of Table 4, there is a significant difference in the mental wellbeing levels among career women with different childbirth statuses. There may be two reasons: firstly, most of women's time may be occupied taking care of two children. It limits their flexibility in the work and affects their career development, leading to job insecurity, which negatively impacts their mental wellbeing. Secondly, two children require more attention and care, continuously draining a woman's time and energy besides work. This makes it difficult for them to get adequate relaxation. Consequently, accumulated exhaustion and psychological stress can result in mental wellbeing issues.

**Table 4:** Mental wellbeing status of career women with different childbirth status.

**Statistical content**	**Variables**	**Number**	**Portion of positive test results**	**Positive rate of depression**	**Positive rate of anxiety**	**Chi-square test**		
						**DF**	**Chi-value**	***P*** **-value**
Childbirth status	**Childless**	225	40.88%	41.33%	20.44%	2	9.427	0.009
	Women with one **children**	383	30.26%	32.11%	16.71%			
	Women with two **or more children**	80	42.50%	43.75%	25.00%			

### 4.2 The perceived adverse impact of childbirth on the working environment may worsen career women's mental wellbeing

Combining the items on working environment in Section 3 with mental wellbeing status data in Section 2, a comparative analysis can be conducted between career women with and without children. This analysis explores how the perception of childbearing affects the existing working environment and may further worsen the mental wellbeing of career women.

#### 4.2.1 The perceived adverse effect of childbearing on the working environment negatively affects the mental wellbeing of career women

According to the survey results shown in Table 5, for the item 1 “Does childbearing negatively impact career development?”, 48.71% of childless career women who answered affirmatively showed positive test results, which is higher than that of those who answered negatively at 32.40%. This indicates that the proportion of mental wellbeing issues is higher among childless career women who perceive that childbearing negatively impacts career development compared to those who do not. There may be a correlation between the perceived adverse impact of childbearing on the working environment and the mental wellbeing status of childless career women. The study posed a similar question to career women with children. The survey results for item 1 showed that 50.97% of women who agreed had positive test results, which is higher than the 19.34% of those who disagreed. This evidence provides a definitive “yes” answer to the second item posed by this study: “Are the adverse effects of population decline on the working environments of career women contributing to the deterioration of their mental wellbeing?”

**Table 5:** Survey on the impact of childbearing on the working environment and mental wellbeing status of career women.

**Num**.	**Childbirth status**	**Items**	**Answer**	**Portion of positive test results**
1	Women without children	Assuming you have plans to have children, do you think this would negatively impact your career development?	Yes	48.71%
			No	32.40%
	Women with children	Has having children negatively impacted your career development?	Yes	50.79%
			No	19.34%

Further investigation reveals that childbearing negatively impacts the working environment in several ways, including “reduced promotion opportunities,” “lower income,” “downward job transfers,” and “forced career interruptions,” as shown in Table 6. Regardless of whether career women have children or not, those who perceive that childbearing negatively impacts the working environment show a higher rate of mental wellbeing issues than those who disagree. This further supports the statement: “The perceived adverse impact of childbearing on the working environment is one of the factors contributing to the deterioration of career women's mental wellbeing.”

**Table 6:** Survey on the specific impact of childbearing on the working environment and mental wellbeing status of career women.

**Num**.	**Childbirth status**	**Items**	**Answer**	**Portion of positive test results**
2	Women without children	Assuming you have plans to have children, do you think this would make it difficult for you to find a desirable job?	Yes	48.80%
			No	31.00%
	Women with children	Has having children made it difficult for you to find a desirable job?	Yes	51.81%
			No	26.06%
3	Women without children	Assuming you have plans to have children, do you think this would reduce your promotion opportunities?	Yes	44.77%
			No	35.16%
	Women with children	Has having children reduced your promotion opportunities?	Yes	50.35%
			No	24.38%
4	Women without children	Assuming you have plans to have children, do you think this would reduce your training opportunities?	Yes	44.28%
			No	35.29%
	Women with children	Has having children reduced your training opportunities?	Yes	57.82%
			No	22.83%
5	Women without children	Assuming you have plans to have children, do you think this would reduce your income?	Yes	45.38%
			No	34.73%
	Women with children	Has having children reduced your income?	Yes	45.02%
			No	24.65%
6	Women without children	Assuming you have plans to have children, do you think this would force you to take a demotion?	Yes	52.38%
			No	34.04%
	Women with children	Has having children forced you to take a demotion?	Yes	52.50%
			No	27.93%
7	Women without children	Assuming you have plans to have children, do you think this would make you interrupt your career?	Yes	45.34%
			No	38.12%
	Women with children	Has Having Children made you interrupt your career?	Yes	44.20%
			No	27.07%

#### 4.2.2 Childless career women are overly concerned about the negative impact of childbearing on career development

Compared to career women with children, childless career women have stronger concerns about the negative impact of childbearing on career development and exhibit lower psychological resilience. As shown in Table 7, 52% of childless career women agreed that “childbearing negatively impacts career development,” whereas 40.82% of career women with children shared this view. The difference between the two groups indicates that childless career women are more worried about the negative impact of childbearing on their career development and have weaker stress-coping abilities compared to their counterparts with children.

**Table 7:** Survey on the specific impact of childbearing on the working environment.

**Num**.	**Childbirth status**	**Items**	**Answer**	**Number**	**Proportion**
1	Women without children	Assuming you have plans to have children, do you think this would negatively impact your career development?	Yes	117	52.00%
		Women with children	Has having children negatively impacted your career development?	Yes	189	40.82%
2	Women without children		Assuming you have plans to have children, do you think this would make it difficult for you to find a desirable job?	Yes	125	55.56%
		Women with children	Has having children made it difficult for you to find a desirable job?	Yes	110	23.76%
3	Women without children		Assuming you have plans to have children, do you think this would reduce your promotion opportunities?	Yes	134	59.56%
		Women with children	Has having children reduced your promotion opportunities?	Yes	139	30.02%
4	Women without children		Assuming you have plans to have children, do you think this would reduce your training opportunities?	Yes	140	62.22%
		Women with children	Has having children reduced your training opportunities?	Yes	174	37.58%
5	Women without children		Assuming you have plans to have children, do you think this would reduce your income?	Yes	130	57.78%
		Women with children	Has having children reduced your income?	Yes	171	36.93%

Subsequent survey results further confirm this point. For the statements that childbearing would “make it difficult for career women to find desirable jobs,” “reduce promotion opportunities,” “reduce training opportunities,” and “reduce labor income,” the percentages of childless career women who agreed were 55.56%, 59.56%, 62.22%, and 57.78%, respectively. These percentages are higher than those of career women with children, who agreed at rates of 23.76%, 30.02%, 37.58%, and 36.93%, respectively. The reasons for these survey results may primarily be attributed to two factors. Firstly, after giving birth, career women take on the role of “mother,” which enhances their sense of responsibility and self-worth. This transformation boosts their psychological resilience and alleviates depression and anxiety among women with children. Secondly, digital technology has led to the rapid development of social media, which has amplified the fear of childbirth among childless career women. Childbearing itself is a concern for these women, and the personalized algorithmic recommendations on social platforms have further deepened their fears and negative emotions regarding childbirth (43). Additionally, the current economic instability has also affected the mental wellbeing of childless career women by fostering a pessimistic attitude toward their employment prospects. Most childless career women are in the early stages of their careers, and according to the research by Li et al. (44) and Yan et al. (45), there is a close correlation between optimism about employment prospects and the presence of depressive symptoms.

### 4.3 The perceived adverse impact of childbirth on the family environment may worsen the mental wellbeing of career women

Combining the items on family environment in Section 3 with mental wellbeing status data in Section 2, a comparative analysis can be conducted between career women with and without children. This analysis explores how childbearing affects the existing family environment, further worsening the mental wellbeing of career women.

#### 4.3.1 The perceived adverse impact of childbearing on the family relationships and its potential effects on the mental wellbeing of career women

According to the data in Table 8, this survey found that “the adverse impact of childbearing on family relationships worsens the mental wellbeing of career women.” From Table 8, the survey results for item 1 indicate that a higher portion of positive test results was observed among childless career women who agreed with this statement, at 47.82%, compared to those who disagreed, at 37.82%. It suggests that the negative impact of childbearing on family relationships is likely to cause mental wellbeing problems for career women. The survey results for career women with children also support this conclusion. The highest portion of positive test results was observed among those who agreed with the statement, at 65.42%, which is higher than that of those who disagreed.

**Table 8:** Survey on the impact of childbearing on family relationships.

**Num**.	**Childbirth status**	**Items**	**Answer**	**Portion of positive test results**
1	Women without children	Assuming you have plans to have children, do you think this would negatively impact your family relationships?	Yes	47.82%
			No	37.82%
	Women with children	Has having children negatively impacted your family relationships?	Yes	65.42%
			No	22.19%
2	Women without children	Assuming you have plans to have children, do you think this would affect your marital relationship?	Yes	49.43%
			No	35.29%
	Women with children	Has having children affected your marital relationship?	Yes	63.04%
			No	24.52%
3	Women with children	Have you had conflicts with their first child due to having multiple children?	Yes	50.70%
			No	28.82%

According to the data from Item 2 and Item 3 in Table 8, 49.43% of childless career women who believe that childbearing affects marital relationships had positive test results. Similarly, 63.04% of career women with children who hold the same belief had positive test results. These rates are higher than those of women who disagreed. This implies that discord in marital relationships could have a detrimental effect on the mental wellbeing of career women. According to previous literature, marital status has an impact on women's mental health, with harmonious marriages offering a protective effect that can effectively improve women's mental wellbeing (46, 47). Additionally, 50.70% of career women who reported experiencing family conflicts with their first child, possibly as a result of having multiple children, showed positive test results. This rate is higher than that of those who did not report such conflicts. This suggests that conflicts with the first child arising from having additional children are likely to worsen the mental wellbeing of career women.

#### 4.3.2 The increased household responsibilities from childbearing worsen the mental wellbeing of career women

The analysis of Tables 9–12 reveals that most career women agree that “childbearing increases household responsibilities,” including housework, childcare, and financial burdens. Moreover, career women who affirm that “childbearing increases household responsibilities” tend to have poorer mental wellbeing compared to those who disagree. This suggests that the increased household responsibilities associated with childbearing may negatively impact the mental wellbeing of career women.

**Table 9:** Survey on the specific impact of childbearing on family responsibility.

**Num**.	**Items**	**Answer**	**Proportion**
4	Do you think childbearing is a family responsibility that women must bear?	Yes	24.71%
		No	75.29%
5	Have you experienced pressure from your spouse or older adults to have children	Yes	20.64%
		No	79.36%

According to the responses to Items 4 and 5 in Table 9, this study finds that social conceptions in China are beginning to change, with most people believing that childbearing is not necessarily a responsibility that women must bear. For Item 4, “Do you think childbearing is a family responsibility that women must bear?”, 24.71% of career women agreed, while 75.29% disagreed. The results for Item 5, “Have you experienced pressure from your spouse older adults to have children?” show that 79.36% of career women have not experienced such pressure. However, according to the survey results in Tables 10–12 regarding the specific household responsibilities undertaken by career women, women still bear the majority of household care responsibilities. The traditional belief that “men bring home the bacon, women take care of the house”^4^ remains deeply rooted in China (48).

**Table 10:** Survey on the specific impact of childbearing on household labor responsibilities.

**Num**.	**Childbirth status**	**Items**	**Answer**	**Proportion**	**Portion of positive test results**
**6**	Women without Children	Assuming you have plans to have children, do you think this will make you take on more household chores in the future?	Yes	56.89%	51.56%
			No	43.11%	26.80%
	Women with Children	Has having children caused you to take on more household chores?	Yes	54.86%	42.51%
			No	45.14%	19.61%

The results from Table 10 indicate that childbearing increases the household labor responsibilities of career women, thereby worsening their mental wellbeing. Over 50% of career women answered yes to Item 6, “Does childbearing make you responsible for more household chores?” Among those who agreed, about 50% experienced mental wellbeing issues. In contrast, the lower portion of positive test results was observed among childless career women at 26.80% and among career women with children at 19.61%, both of whom disagreed with this statement, lower than those who agreed. In today's society, women's roles are often stereotypically seen as those of mothers and wives, confining them to household chores that neither reflect their personal value nor their societal contribution. Statistics show that Chinese women spend 2.6 times more time on unpaid household labor than men (49). Globally, 75% of unpaid work is done by women, who spend 3 to 6 h a day on such tasks, while men spend an average of 30 min to 2 h (50). The extended hours of unpaid household chores make it difficult for women to achieve a sense of self-actualization, leading to a higher likelihood of mental wellbeing issues among career women over time.

The results from Table 11 indicate that childbearing increases the responsibilities of career women in caring for young children, thereby worsening their mental wellbeing. For Item 7, “Do you think childbearing will lead to more childcare responsibilities?”, 64.44% of childless career women and 69.33% of career women with children responded affirmatively. Among those who agreed, the higher portion of positive test result was observed among childless women at 48.96% and among women with children at 39.25%, both higher than those who disagreed. This shows that childbearing increases the childcare responsibilities of career women, leading to mental wellbeing issues. In China, many career women often face the challenge of “single-handed parenting,” where the father is not involved in day-to-day childcare, leaving the mother to handle the majority of the responsibilities alone. Unlike ordinary women, career women with higher education and income levels also face greater work-related pressure. The burden of “single-handed parenting” impacts their mental wellbeing, causing severe psychological issues. After a day of high-pressure work, career women must undertake a “second shift” at home to care for their children, depleting their already limited energy and subjecting them to intense mental strain (51). Additionally, most career women today were born after China's implementation of the One-child Policy. As a result, most families have only one child who receives the most attention and best resources from the entire family. Especially in the case of only daughters, they are often spoiled as the “princess” of the family. However, after marriage, these women must take on the responsibility of caring for four older adults in addition to raising their own children. This significant role shift forces women to shoulder a heavy household burden, resulting in substantial psychological discrepancies. The increased household responsibilities and psychological gaps intensify the stress on career women, leading to more severe mental wellbeing issues (52).

**Table 11:** Survey on the impact of childbearing on responsibilities in caring for young children.

**Num**.	**Childbirth status**	**Items**	**Answer**	**Proportion**	**Portion of positive test results**
**7**	Women without children	Assuming you have plans to have children, do you think this will make you take on more childcare responsibilities in the future?	Yes	64.44%	48.96%
			No	35.56%	26.25%
	Women with children	Has having children caused you to take on more childcare responsibilities?	Yes	69.33%	39.25%
			No	30.67%	16.19%

Table 12 shows whether childbearing increases the economic burden on career women. The results align with expectations, showing that childbearing indeed exacerbates the economic burden on career women, thereby worsening their mental wellbeing. Among career women, 64.89% of those without children and 63.93% of those with children agreed with this statement. The higher portion of positive test results was observed among childless women at 47.94% and among women with children at 42.56%, higher than the portion for those who disagreed, which were 27.84% and 13.06%, respectively. This indicates that the increased economic burden from childbearing is likely to trigger mental wellbeing issues in career women. In today's climate of increased economic uncertainty, career women need to share financial responsibilities with men. When children are 0–3 years old, career women often have to devote most of their energy to childcare, sometimes even quitting their jobs to become full-time housewives. This career interruption can hinder their professional development. In today's competitive employment market, extended periods of absence can heighten reemployment difficulties among career women. Additionally, this period is typically when families face the greatest pressure from mortgage and car loan payments. If women quit their jobs, it cuts off a significant source of household income. Men have to shoulder the heavy financial burden alone. This increases men's anxiety. The accumulated stress can lead to frequent arguments between spouses, further exacerbating women's anxiety and depression.

**Table 12:** Survey on whether childbearing increases the economic burden on career women.

**Num**.	**Childbirth statuses**	**Items**	**Answer**	**Proportion**	**Portion of positive test results**
8	Women without children	Assuming you have plans to have children, do you think this will increases your economic burden?	Yes	64.89%	47.94%
			No	35.11%	27.84%
		Women with children	Has having children increased your economic burden?	Yes	63.93%	42.56%
	No			36.07%	13.06%

### 4.4 Current policy satisfaction and mental wellbeing needs of career women

#### 4.4.1 Lack of awareness about women's rights protection policies leads to no satisfaction among career women

At present, the “Three-Child Policy” has been fully implemented, leading more career women to face the question of “how many children to have.” This increases the pressure on career women both in the workplace and at home. Women's rights protection policies, as the most important means of safeguarding their rights in both the workplace and the home, are neither well-known nor satisfactory to many career women. This lack of awareness and satisfaction is a significant factor contributing to the anxiety and depression experienced by career women (53). Before investigating career women's satisfaction with current policies and their mental wellbeing needs, this study first surveyed their awareness of women's rights protection policies. From Table 13, the survey results show that only 25.44% of career women are aware of local or organization-specific policies on protecting women's special rights. Regarding the policies protecting the specific rights related to women's childbirth, only 25.73% of career women are aware, while 74.27% are unaware. The survey also reveals that only 30.08% of career women are satisfied with the current women's rights protection policies. Additionally, 44.76% of career women are satisfied with their current organization's efforts to protect women's rights. The majority of career women do not feel satisfied with the current women's rights protection policies or their organization's efforts in this regard.

**Table 13:** Survey on career women's awareness and satisfaction with women's rights protection policies.

**Statistical content**	**Items**	**Answer**	**Proportion**
Awareness	Are you aware of local or organization-specific women's rights protection policies^1^?	Very understandable	8.58%
		Understandable	16.86%
		General	41.86%
		Un-understandable	24.71%
		Completely un-understandable	7.99%
	Are you aware of local policies specifically protecting the rights of women having a second or third child^2^?	Very understandable	8.14%
		Understandable	17.59%
		General	36.19%
		Un-understandable	29.8%
		Completely un-understandable	8.28%
Satisfaction	Are you satisfied with the current women's rights protection policies?	Very satisfied	7.99%
		Satisfied	22.09%
		General	54.07%
		dissatisfied	10.90%
		Completely dissatisfied	4.95%
	Are you satisfied with your organization's efforts to protect women's rights?	Very satisfied	11.77%
		satisfied	32.99%
		General	43.46%
		dissatisfied	7.12%
		Completely dissatisfied	4.66%

1. Local policies for screening and treatment of gynecological diseases, such as cervical cancer and breast cancer; local community policies on the distribution of hygiene products; and organization policies on the protection of women's special rights.

2. The specific number of days for maternity leave, breastfeeding leave, childcare leave, and paternity leave in the local area, as well as whether the number of leave days differs for the first, second, and third child. For example, in a certain province, maternity leave is 158 days for the first child and 188 days for the second and third child; the distribution of maternity benefits and salaries during maternity leave, and whether the maternity benefits differ for the first, second, and third child. For example, in a certain city, a one-time subsidy of 2,000 RMB is provided for the second child, and a one-time subsidy of 5,000 RMB is provided for the third child.

#### 4.4.2 Career women's needs primarily focus on reducing family burden

As shown in Table 14, the survey finds that career women's needs are largely focused on reducing family responsibilities and alleviating the stress caused by these responsibilities. Within the family, most career women believe that “sharing childcare duties among family members,” “actively participating in household chores,” and “sharing the economic burden” are effective measures to alleviate mental wellbeing issues. This aligns with previous findings that childbearing increases family responsibilities for career women and supports the conclusion that “the mental wellbeing issues of career women are related to family responsibilities.” In the workplace, the top three needs of career women are “flexible working hours,” “reasonable bonuses and subsidies,” and “equal promotion opportunities for men and women,” accounting for 71.80%, 58.28%, and 49.13% of the total participants, respectively. At the same time, options specifically aimed at protecting women's special rights, such as “equal opportunities for vocational skills training,” “consideration of women's participation in team-building activities,” “construction of female-specific facilities,” “mental wellbeing training,” and “provision of essential female hygiene products in restrooms,” were considered necessary by only a small portion of career women. On a societal level, the top three needs for career women are “extended maternity leave, spouse paternity leave, and childcare leave,” “provision of maternity and childcare subsidies,” and “establishment of professional childcare facilities.”

**Table 14:** Survey on career women's mental wellbeing needs.

**Statistical content**	**Items**	**Proportion**
Needs from family	Sharing childcare duties among family members	70.78%
	Actively participating in household chores	67.01%
	Sharing the economic burden	57.56%
	Understanding each other	39.1%
	Childbearing decisions are made jointly by the couple	14.39%
	Learning understanding mental wellbeing knowledge together	13.81%
	Others	3.78%
Needs from workplace	Flexible working hours	71.80%
	Reasonable bonuses and subsidies	58.28%
	Equal promotion opportunities for men and women	49.13%
	Effective communication mechanism	29.36%
	Equal opportunities for vocational skills training	13.52%
	Consideration of women's participation in team-building activities	11.48%
	Construction of female-specific facilities	10.03%
	mental wellbeing training	8.87%
	Provision of essential female hygiene products in restrooms	7.70%
	Others	3.34%
Needs from society	Extended maternity leave, spouse paternity leave, and childcare leave	67.01%
	Provision of maternity and childcare subsidies	62.35%
	Establishment of professional childcare facilities	42.88%
	Strengthen the publicity of mental wellbeing	26.60%
	Organize lectures related to women's reproductive health	24.27%
	Offering employment guidance and training	19.33%
	Establish mental wellbeing counseling rooms in the community	11.19%
	Others	4.65%

The mental wellbeing needs of career women are mainly focused on reducing family responsibilities. This indicates that the causes of their mental wellbeing issues stem more from the family environment rather than the workplace. Within the family, career women hope that family members can share household responsibilities instead of adhering to the traditional notion of “men bring home the bacon, women take care of the house.” In the workplace, the need for flexible working hours largely arises from the necessity to better care for the family, reflecting the reality that many career women still prioritize the belief that “women should prioritize the family.” The coexistence of these two beliefs within the same individual fully reflects the current self-contradictory mindset of career women. On one hand, they want to break free from the traditional notion, but on the other hand, they still take on most of the family responsibilities themselves.

## 5 Discussion

**Figure 1:**
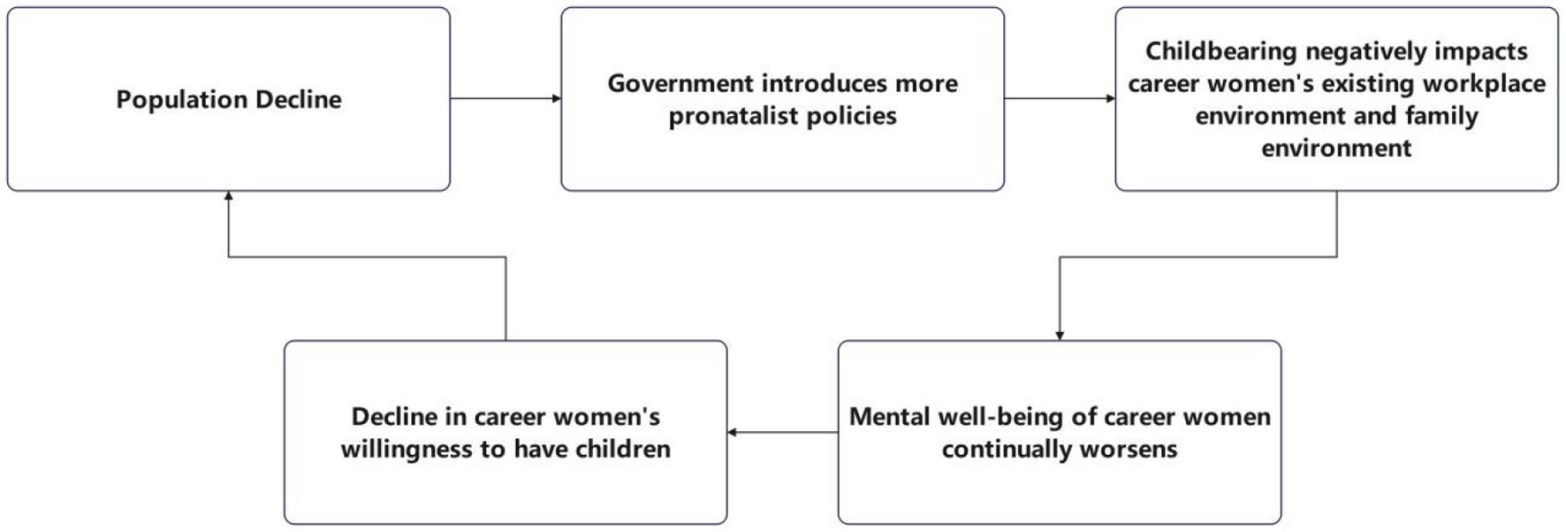
The vicious cycle of population decline.

As shown in Figure 1, the pro-natalist policies introduced in response to population decline can worsen the mental wellbeing of career women, while the deterioration of their mental wellbeing can further accelerate the trend of population decline. Currently, the “Three-Child Policy” in China shifts the focus for career women from “whether to have children” to “how many children to have.” This requires women to devote substantial time and energy to pregnancy and child-rearing, which increases the costs for companies employing female employees (54). Consequently, this leads to a decrease in companies' willingness to hire female employees and exacerbates work discrimination (55). At the same time, as analyzed above, although women now have much greater freedom in deciding “whether to have children,” traditional family values remain deeply entrenched. Career women still lack options when it comes to deciding “whether to raise children” or “who should take on more family responsibilities.” They often have no choice but to compromise (56). Having multiple children increases the family responsibilities of career women, further reducing their willingness to have children (57). In response to population decline, the government is likely to introduce more pro-natalist policies, creating a vicious cycle that negatively impacts the mental wellbeing and fertility intentions of career women (58).

### 5.1 Challenges

However, it is important to note that several challenges should be discussed. First, how can the fear of childbearing among childless career women be alleviated, and their psychological resilience enhanced? The survey results indicate that 83.55% of childless career women are under the age of 35, suggesting a high potential for fertility. Therefore, it is necessary to address their concerns about the negative impact of childbearing on career development and to improve the willingness of this group to have children. This can not only enhance the overall mental wellbeing of women in China but also help increase the birth rate and support the implementation of the “Three-Child Policy.” Second, how can the family responsibilities of career women be reduced? While the economic burden of the family is generally shared by both spouses, the responsibilities of household labor and childcare are often primarily or solely borne by women. Therefore, it is crucial to encourage spouses to share these responsibilities. Third, how can the awareness and satisfaction with women's rights protection policies among career women be improved? Currently, with the full implementation of the “Three-Child Policy,” the number of families having a second or third child is on the rise. This increases the pressure on career women both in the workplace and at home. However, it is concerning that career women generally have low levels of awareness and satisfaction with women's rights protection policies, which is a key factor contributing to their anxiety and depression.

### 5.2 Recommendations

First, it is crucial to focus on the psychological wellbeing of childless career women. From the individual perspective career women should maintain a healthy lifestyle, including a balanced diet, regular exercise, and adequate sleep. This helps to relax the body and mind, thereby alleviating stress (59). Additionally, career women should actively learn about laws, regulations, rules, and other normative documents related to women's rights, to increase their awareness of their rights and know how to protect their legal rights. From the corporate perspective, the concerns of childless career women often stem from work discrimination against pregnant employees. Companies should rigorously implement women's rights protection policies, strictly adhere to relevant labor regulations, and avoid covert gender discrimination. Additionally, companies should provide humane care by establishing nursing rooms and improving the work environment for women. At the societal level, the government can actively organize lectures related to women's reproductive health, enriching career women's theoretical knowledge in this area. Childless career women can benefit from scientific knowledge about the reproductive process, prenatal care, and postnatal recovery through expert lectures, online resources, and books, thereby eliminating misconceptions and fears about childbirth. From a societal perspective, the government can actively organize lectures on women's reproductive health to enrich the theoretical knowledge of career women. Through expert lectures, official online resources, and related books, childless career women can gain scientific knowledge about the reproductive process, prenatal care, and postnatal recovery, thereby eliminating misconceptions and fears about childbirth.

Second, it is necessary to reduce the burden of family responsibilities on career women. The first and most important step is to change the traditional notion of “men bring home the bacon, women take care of the house” in society. This can be achieved by distributing informational pamphlets in communities and organizing public lectures to enhance men's awareness of family responsibilities. Men should recognize that women's needs are not only material but also include psychological companionship and emotional support. Furthermore, women need to liberate themselves from the traditional mindset of being solely responsible for household duties. In families, women often take on most, if not all, household chores and childcare responsibilities, either voluntarily or involuntarily. Over time, this creates a habitual mindset in men that these responsibilities inherently belong to women, which is detrimental to the mental wellbeing of career women. Cooperation between men and women in household chores and childcare not only improves family happiness but also benefits the development of children (60). Therefore, women should actively involve their spouses in sharing family responsibilities, especially in childcare. If women shoulder too much of the childcare burden, it can lead to the absence of a father figure in the child's development, negatively impacting the child's growth. Finally, the country should vigorously develop childcare facilities, establish professional day-care centers, and improve public services to alleviate the family burdens on career women. There should be increased regulation and strict supervision of childcare facilities and personnel. Currently, one of the main reasons for increased childcare pressure on women is the lack of professionalism in childcare facilities. Frequent reports of child abuse and mistreatment force women to invest excessive energy in childcare.

Third, it is practical to continuously improve policies and laws protecting the rights of career women. To begin with, digital information technology and social media platforms can be utilized to enhance the promotion of women's rights protection policies and related laws. In the aforementioned survey, most career women were not aware of these policies. Therefore, the government can use internet platforms such as WeChat, TikTok, and Weibo to publicize these policies, thereby increasing awareness among career women. Furthermore, the legal framework for protecting women's rights should be improved. Women should be encouraged and given more opportunities to engage actively in political activities. This will enhance their representation and voice, enabling the country and society to understand the needs and challenges faced by career women. As a result, relevant policies can be formulated to address these issues and protect the rights of career women. Finally, women's rights protection agencies should be established to encourage women to participate in safeguarding their rights. These agencies should provide legal consultation and assistance services to women, empowering them and ensuring they have access to the support needed to uphold their rights.

## 6 Conclusion

This study provides a comprehensive analysis of the mental wellbeing of career women in the context of China's population decline, focusing on the perceived impact of both working and family environments on their mental wellbeing. The results from the chi-square tests indicate significant differences in mental wellbeing across different age groups, industries, and childbirth statuses. Specifically, career women in the middle-aged group, those working in education, healthcare, and machinery manufacturing, and those with two children exhibit higher levels of mental wellbeing issues. These findings underscore the importance of considering demographic and occupational factors when addressing the mental health challenges faced by career women. In addition to these demographic and occupational factors, the study also highlights the perceived adverse impacts of childbirth on career development and family relationships as significant contributors to mental health challenges among career women. These perceived impacts, although not definitive, suggest that the dual pressures from professional responsibilities and family obligations may exacerbate mental wellbeing issues. Furthermore, the research indicates that career women are not satisfied with the current policies protecting women's rights, particularly in relation to pro-natalist policies. These policies, while intended to encourage childbirth, may inadvertently exacerbate the challenges faced by career women, especially in balancing work and family life. There seems to be a clear need for comprehensive policies that reduce family responsibilities, enhance workplace flexibility, and improve awareness of women's rights protection. Given these findings, it is essential to consider a multifaceted approach involving both individual and societal interventions to address the mental wellbeing challenges faced by career women. Encouraging greater involvement of men in household duties, improving childcare facilities, enhancing workplace flexibility, and reinforcing legal protections for women in the workforce are vital steps, among others. These measures should be supported by ongoing research and data collection to monitor their effectiveness and to adapt policies as needed. By addressing these areas comprehensively, not only can the mental health of career women be significantly improved, but these efforts may also contribute to creating a more supportive environment for women, potentially reversing the negative trends in population growth.

### 6.1 Limitations

Although this study expands upon previous research, it still has certain limitations. First, this study did not include another significant group of women—non-career women. In the context of population decline, pro-natalist policies may have a considerable impact on this group. Since non-career women do not need to work outside the home, they may be expected to take on more family responsibilities, including childbirth. However, career women and non-career women differ significantly in their social roles, responsibilities, and sources of stress. Combining these two groups in a single study could lead to confounded results that do not accurately reflect the unique mental health challenges faced by career women. Therefore, future research should further focus on non-career women as a distinct group to enhance the depth and scope of the research. Second, the study is geographically limited to China, so the findings and proposed solutions may not be applicable to countries and regions with significantly different population structures and levels of economic development. Additionally, while the study compares the mental wellbeing of career women with and without children, it is important to acknowledge that the responses from childless women are based on perceived impacts, which may differ from the actual experiences of women with children. Therefore, future research could include longitudinal studies on childless women to better understand the evolution of their perceptions and experiences over time.

## Data Availability

The original contributions presented in the study are included in the article/Supplementary material, further inquiries can be directed to the corresponding author.

## Footnotes

^1^This data is sourced from the Population Division of the United Nations (https://population.un.org/wpp/).

^2^This data is sourced from the National Bureau of Statistic of China (https://www.stats.gov.cn/english/).

^3^This data is sourced from the *Green Paper on the Mental Well-Being of Chinese Career Woman, 2019* (https://www.blackmoresinstitute.org/-/media/bklbi/pdf/2019-green-paper-on-the-mental-wellbeing-of-chinese-career-women.pdf).

^4^This phrase is a local expression reflecting traditional gender roles, deeply rooted in Confucian thought in China, where men were seen as the providers and women as the caretakers of the household.

## References

[1] Wang QC. A summary of population thought of communist party of China Wang Qin-chi. Northwest Popul J. (2022) 43:14–24. 10.15884/j.cnki.issn.1007-0672.2022.06.002

[2] Nguyen VK Lock MM. An Anthropology of Biomedicine. New York, NY: John Wiley & Sons Ltd. (2011).

[3] Chen JH Mao KY Meng FC Liu HJ. China's high-quality population development: connotation, level and regional differences. Chin J Popul Sci. (2024) 38:3–18.

[4] Shi ZL Yang YZ. Are People with Higher Education More Li-kely to Get Depression? The effect of education on adults' depressive symptom-s. J Beijing Normal Univer. (2020) 2:148–60.

[5] Lindebaum D. Does emotional intelligence moderate the relationship be-tween mental health and job performance? An exploratory study. Eur Managem J. (2012) 31:538–48. 10.1016/j.emj.2012.08.002

[6] Wang L. A study on the characteristics, trend, and problems of family structure changes in China: based on the analysis of the national census micro-data. J Peking Univer. (2024) 61:140–51.

[7] Feng DD Lu CQ Xiao AL. Job insecurity, wellbeing, and job performance: the role of general self-efficacy. ActaPsychologica Sinica. (2008) 40:448–55. 10.3724/SP.J.1041.2008.0044837113526

[8] Li QM Wang JT. The relationship between family atmospher-e children's life satisfaction and intergenerational transmission of mental healt-h: the difference of parent-child gender and children age. Stud Psychol Behav. (2022) 20:282–8. 10.12139/j.1672-0628.2022.02.020

[9] Yang ZH. Preliminary analysis of negative population growth in Taicang county. Populat Res. (1985) 3:36–40. 12341126

[10] Huang GY. The impact of population regeneration cycles on negative population growth in Taicang County in 1983 and 1984. Chin J Health Statist. (1988) 3:64.

[11] Wang F Guo ZG Mao ZY. A preliminary study of China'-s negative population growth momentum in the 21st century. Popul Res. (2008) 32:7–17.

[12] Coleman D Rowthorn R. Who's afraid of population decline? A critical examination of its consequences. Popul Dev Rev. (2011) 37:217–48. 10.1111/j.1728-4457.2011.00385.x 21280372

[13] Zhang XL Zhai ZW Tao T. Trends and patterns of negative population growth in China. Populat Res. (2020) 44:3–20.

[14] Jin GZ Guo YL Tao T. Negative population growth: conceptual evolution, multidimensional classification, and economic impacts. Lanzhou Acad J. (2024) 2:64–76.

[15] Zheng GH. Role conflict and depression prevention of professional women under the background of the three-child policy. Acad Forum (2022) 45:72–82. 10.16524/j.45-1002.2022.03.005

[16] Chen JY Ma JQ. The Causes and Countermeasures of low birth rates among young people under the Three-Child Policy. China Youth Study. (2022) 3:31–6. 10.19633/j.cnki.11-2579/d.2022.0040

[17] Ye X. Negative population growth in china: urrentsituation, challenges and countermeasures. J Yangzhou Univer. (2023) 27:76–92. 10.19411/j.cnki.1007 7030.2023.05.007

[18] Nordenmark M Almén N Vinberg S. Work/family conflict of more importance than psychosocial working conditions and family conditions for mental wellbeing. Societies. (2020) 10:67. 10.3390/soc10030067

[19] Wang M Wang SC. A Study of Children's Educational Pressure and Housing Pressure on the Fertility Intentions from the Perspective of the Crowding out Effect of Happiness and Social Class. South China Populat. (2024) 39:14–26.

[20] Shi LZ Wang Z. Extended maternity leave and migrant women's fertilityunemployment paradox: based on China migrants dynamic survey data. Chin J Sociol. (2024) 44:213–42. 10.15992/j.cnki.31-1123/c.2024.02.008

[21] Srivastava N Anand M. Understanding gender and mental health. In:Anand M, editor. Gender and Mental Health Combining Theory and Practice, Singapore: Springer. (2020) 3–19. 10.1007/978-981-15-5393-6_1

[22] Wang ZH Yu WL Shen Z Ye Y Hu L Yu GX. Reliability and validity of the symptom checklist 90 in Chinese professional females. Chin J Indust Med. (2017) 30:247–50. 10.13631/j.cnki.zggyyx.2017.04.002

[23] Herrman H Patel V Kieling C Berk M Buchweitz C Cuijpers P. Time for united action on depression: a Lancet-World Psychiatric Association Commission. Lancet. (2022) 399:957–1022. 10.1016/S0140-6736(21)02141-3 35180424

[24] Bacigalupe A Martín U. Gender inequalities in depression/anxiety and the consumption of psychotropic drugs: are we medicalising women's mental health?. Scandinavian J Public Health. (2021) 49:317–324. 10.1177/1403494820944736 32755295

[25] Yan B Gong WT Yao T Liu YC Sun L. Analysis of mental health status of building professional women in Hongqiao Business District of Shanghai. Shanghai Med Pharmaceut J. (2022) 43:14–8.

[26] Lv L Wang KR Li J Guo ZQ Wang DC Li YH. Survey on the psychological health and social support among women of different population in the urban of Ningxia. Ningxia Med J. (2011) 33:421–3. 10.13621/j.1001-5949.2011.05.001

[27] Gao J Jiang LH Ni S Wu HC. Research on the influencing factors and countermeasures of depression among occupational women in functional communities based on IMB structural equation model. Chin J General Pract. (2023) 21:2093–123. 10.16766/j.cnki.issn.1674-4152.003298

[28] Lv CL Lu Y Yan YP Li L Zhong YP. Effect of mental health intervention on WeChat platform on psychological stress of professional women during pregnancy. China Modern Med. (2023) 30:680–83+87.

[29] Adams RE Hu YR Figley CR Urosevich TG Hofman SN Kirchner HL. Risk and protective factors associated with mental health among female military veterans: results from the veterans' health study. BMC Women's Health. (2021) 21:55. 10.1186/s12905-021-01181-z 33557798 PMC7869200

[30] Gavin JF. Employee perceptions of the work environment and mental health: a suggestive study. J Vocat Behav. (1975) 6:217–34. 10.1016/0001-8791(75)90048-2

[31] Travasso SA Rajaraman D Heymann SJ. A qualitative study of factors affecting mental health amongst low-income working mothers in Bangalore, India. BMC Women's Health. (2014) 14: 1–11. 10.1186/1472-6874-14-22 24502531 PMC3922014

[32] Guo L Mao CG. (2023). The gender difference in income distribution –explanations from wage and pension. Soft Sci. 32:129–32. 10.13956/j.ss.1001-8409.2018.09.28

[33] Wang YF. Analysis of conflicts between women's work and family under the comprehensive two child policy. Huxiang Forum. (2017) 30:126–31. 10.16479/j.cnki.cn43-1160/d.2017.06.020

[34] Zhang YM. Comprehensive management of occupational difficulties faced by women of childbearing age under the Two-Child policy. Governance Stud. (2020) 32:129–32. 10.13956/j.ss.1001-8409.2018.09.28

[35] Prel JD Peter R. Work-family conflict as a mediator in the association between work stress and depressive symptoms: cross-sectional evidence from the German lidA-cohort study. Int Arch Occup Environ Health. (2015) 88:359–68. 10.1007/s00420-014-0967-0 25069900

[36] Sun LG Wang ZH Yu WL Shen Z Yu CY Li XF. Study on reliability and validity of a Simplified Scale for Depression and Anxiety Screening among professional females in six industries. Occup Health & Emerg Rescue. (2024) 42:21–5. 10.16369/j.oher.issn.1007-1326.2024.01.004

[37] Gu YT Kou YT Yu CY. Survey on anxiety and depression tendency and influencing factor analysis among female managers. Indust Health Occup Dis. (2024) 50:213–218+257. 10.13692/j.cnki.gywsyzyb.2024.03.00438101214

[38] Li Q. Study on Spatiotemporal Dynamic and Determinants of the Subjective Wellbeing of Married Women in China (Master's thesis). Hangzhou (ZJ): University of Zhejiang. (2022).

[39] Zhang SW Guo FM. (2009). Gender wage discrimination at quantiles:evidence from Northeast Urban Labor market in China. Chin J Popul Sci. 6:69–79+112.

[40] Guo PP Gao K Jiang MM Wang DW. Anxiety and relation with work family conflict and perceived social support in college teachers. Chin Ment Health J. (2023) 37:610–5. 10.3969/j.issn.1000-6729.2023.07.011

[41] Capone V Petrillo G. Mental health in teachers: Relationships with job satisfaction, efficacy beliefs, burnout and depression. Curr Psychol. (2020) 39:1757–66. 10.1007/s12144-018-9878-7

[42] Chen ZW Yu XQ Yu GL. Features, influencing factors, and development trends of mental health problems among secondary school teachers. Renmin Univers China Educ J. (2024) 3:144–55.

[43] Jiang QL Wang HK Chen ZH. Social media information acquisition and its impacts on women's fertility intention: a comparison between information seeking and incidental exposure. Chin J Popul Sci. (2023) 1:39–55.

[44] Li T Zheng YX Yan YT. Have marriage and fertility been de-institutionalized in China?—Findings from a survey on marriage and fertility intentions among college students. J Chin Women's Stud. (2022) 3:85–102.

[45] Yan XJ Chen ZY Xia J FanWJ Zhao XJ. Analysis of influencing factors of depressive symptoms among undergraduate students at a Medical College in Guangzhou. Med Soc. (2024) 37:121–126+144. 10.13723/j.yxysh.2024.03.019

[46] Rutgers DC Vicki A Freedman VA Jennifer C Cornman JC Norbert Schwarz N. Happy marriage, happy life? marital quality and subjective wellbeing in later life. J Marrj Family. (2014) 5:930–48. 10.1111/jomf.12133 25221351 PMC4158846

[47] Wei DX Chen XM. The effect of marriage on personal mental health:an empirical study on china health and retirement longitudinal study in 2011. Northwest Popul J. (2017) 38:103–10. 10.15884/j.cnki.issn.1007-0672.2017.04.014

[48] Shen YF. Super-hot moms: motherhood and women's rights in the individualization era. Nanjing J Soc Sci. (2014) 2:69–77. 10.15937/j.cnki.issn1001-8263.2014.02.013

[49] Lu HN. Reflection on the feminist of the international legal standards of the right to work. Sci Law. (2021) 39:31–42. 10.16290/j.cnki.1674-5205.2021.06.002

[50] Perez CC. Invisible Women. Beijing: New Star Press. (2022).

[51] Xu YT. “Widowed Child-Rearing”: the motherhood dilemma and formation mechanism of the New-Generation urbans mothers. Ningxia Soc Sci. (2020) 6:136–43.

[52] Guo G. Maternal dilemma and gender anxiety in the discourse of ‘widowed child-rearing'. Soc Sci Beijing. (2019) 10:117–28. 10.13262/j.bjsshkxy.bjshkx.191012

[53] Zhang J. Women's fertility penalties in China's labor market. Popul J. (2024) 46:53–66. 10.16405/j.cnki.1004-129X.2024.04.00412316996

[54] Zhang TJ Zhang YJ. A Study on the influence of the universal two-child policy on female employment: based on the mediating effect of enterprise labor cost. Popul Econ. (2017) 5:1–11.

[55] Zhang XX Li Q Gao ZQ. New thoughts on employment discrimination against professional women caused by the universal two-child policy. J Shanxi Univer. (2018) 41:105–13. 10.13451/j.cnki.shanxi.univ(phil.soc.).2018.05.01430329904

[56] Zuo JP. Viewing the inequality between husband and wife in chinese cities from a diversified angle. J Chin Women's Stud. (2002) 1:12–2+13–17.

[57] Li Y Li CA. The impact of gender role attitudes on fertility intentions following the implementation of the universal two-child policy. Northwest Popul J. (2024) 2024:1–15. Available at: http://kns.cnki.net/kcms/detail/62.1019.C.20240612.2229.002.html

[58] Qing SS Wen M. Analysis on the fertility intention for additional births in the three-child policy era: viewing from quantity and certainty perspectives. Chinese J Popul Sci. (2024) 38:82–97.

[59] Firth J Solmi M Wotton RE Vancampfort D Schuch FB Hoare E. A meta-review of “lifestyle psychiatry”: the role of exercise, smoking, diet and sleep in the prevention and treatment of mental disorders. World Psychiatry. (2020) 19:360–80. 10.1002/wps.20773 32931092 PMC7491615

[60] Opondo C Redshaw M Quigley MA. Association between father involvement and attitudes in early child-rearing and depressive symptoms in the pre-adolescent period in a UK birth cohort. J Affect Disord. (2017) 221:115–22. 10.1016/j.jad.2017.06.010 28646709 PMC5523941

